# Data supporting the effects of xanthine derivative KMUP-3 on vascular smooth muscle cell calcification and abdominal aortic aneurysm in mice

**DOI:** 10.1016/j.dib.2020.105550

**Published:** 2020-04-19

**Authors:** Chao-Han Lai, Ching-Wen Chang, Fang-Tzu Lee, Cheng-Hsiang Kuo, Jong-Hau Hsu, Chung-Pin Liu, Hua-Lin Wu, Jwu-Lai Yeh

**Affiliations:** a Department of Surgery, National Cheng Kung University Hospital, College of Medicine, National Cheng Kung University, Tainan, Taiwan; b Cardiovascular Research Center, National Cheng Kung University, Tainan, Taiwan; c Department of Pharmacology, School of Medicine, College of Medicine, Kaohsiung Medical University, 100 Shih-Chuan First Road, Kaohsiung, Taiwan; d Department of Biochemistry and Molecular Biology, College of Medicine, National Cheng Kung University, Tainan, Taiwan; e Department of Pediatrics, College of Medicine, Kaohsiung Medical University, Kaohsiung, Taiwan; f Department of Pediatrics, Kaohsiung Medical University Hospital, Kaohsiung Medical University, Kaohsiung, Taiwan; g Division of Cardiology, Department of Internal Medicine, Yuan's General Hospital, Kaohsiung, Taiwan; h Department of Medical Research, Kaohsiung Medical University Hospital, Kaohsiung, Taiwan; i Department of Marine Biotechnology and Resources, National Sun Yat-sen University, Kaohsiung, Taiwan

**Keywords:** Xanthine derivative KMUP-3, Abdominal aortic aneurysm, *In vitro* calcification, Vascular smooth muscle cell, Phenotypic switch, Apoptosis

## Abstract

No pharmacotherapy in the clinical setting has been available to alter the natural history of abdominal aortic aneurysm (AAA). Targeting vascular smooth muscle cell (VSMC) dysfunction during the pathogenesis of AAA, including phenotypic switch and apoptosis, could be a potential strategy to limit AAA growth. Here, we provide additional information regarding materials, methods and data related to our recent study published in Atherosclerosis [1]. The therapeutic potential of a self-developed xanthine derivative KMUP-3 was evaluated in VSMC calcification and abdominal aortic aneurysm (AAA). *In vitro* VSMC calcification was induced using β-glycerophosphate, and AAA was induced using angiotensin II infusion for 4 weeks in apolipoprotein E-deficient mice. The data contained in this article support the effects of KMUP-3 on VSMC calcification and AAA.

Specifications table

**Table:** 

Subject	Molecular biology.
Specific subject area	Vascular biology.
Type of data	Figures, images and materials and methods.
How data were acquired	Flow cytometry (Coulter Epics XL-MCL), microscope (Carl Zeiss upright microscopes), quantitative real-time polymerase chain reaction (qRT-PCR; StepOne Real-Time PCR System) and western blot analysis.
Data format	Raw and analyzed data.
Parameters for data collection	Phenotypic switch, apoptosis and calcification of cultured vascular smooth muscle cells; medial calcification, inflammatory responses and matrix degradation in the aortic wall.
Description of data collection	The therapeutic potential of KMUP-3 was evaluated in vascular smooth muscle cell (VSMC) calcification and abdominal aortic aneurysm (AAA), and thus samples treated with and without KMUP-3 were collected. VSMC calcification was induced by β-glycerophosphate. The AAA model was induced in apolipoprotein E-deficient mice using angiotensin II infusion.
Data source location	Tainan and Kaohsiung, Taiwan
Data accessibility	Data were included in this article.
Related research article	C.H. Lai, C.W. Chang, F.T. Lee, et al., Targeting vascular smooth muscle cell dysfunction with xanthine derivative KMUP-3 inhibits abdominal aortic aneurysm in mice, Atherosclerosis. 297 (2020) 16–24 [1].

## Value of the data

• The data provides evidence supporting vascular smooth muscle cell dysfunction as a target for treating abdominal aortic aneurysm.
• The data may be used by vascular biologists and other scientists exploring the pathogenesis of vascular calcification and abdominal aortic aneurysm.
• The data may be useful for further investigations regarding vascular calcification, abdominal aortic aneurysm and other vascular diseases.

## 1 Data description

**Fig. 1:**
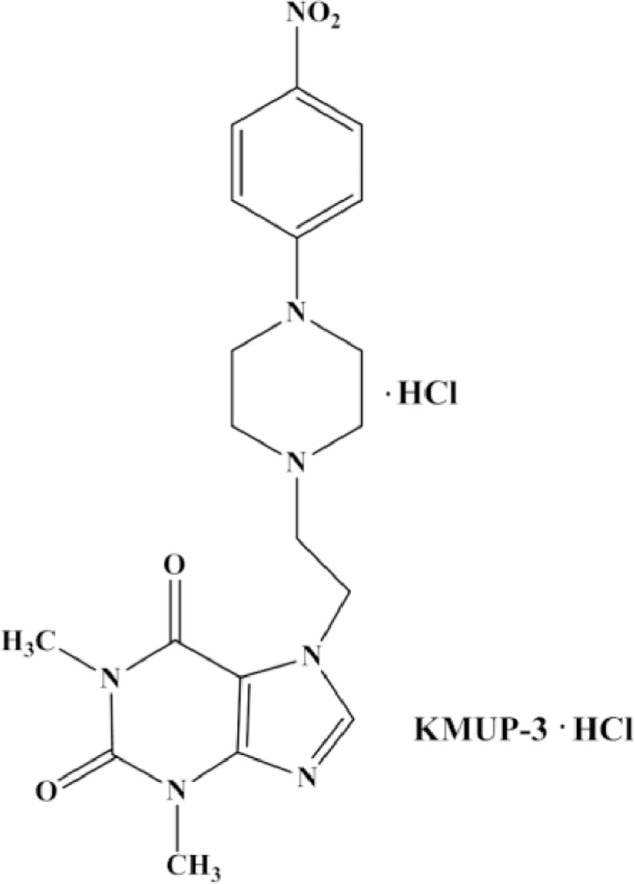
KMUP-3.

The data demonstrates the inhibitory effects of a xanthine derivative 7-[2-[4-(4-*nitrobenzene*)-*piperazinyl*]ethyl]−1,3-dimethylxanthine (KMUP-3; Fig. 1) in vascular calcification and abdominal aortic aneurysm (AAA), serving as supporting data for our recent study [1].

**Fig. 2:**
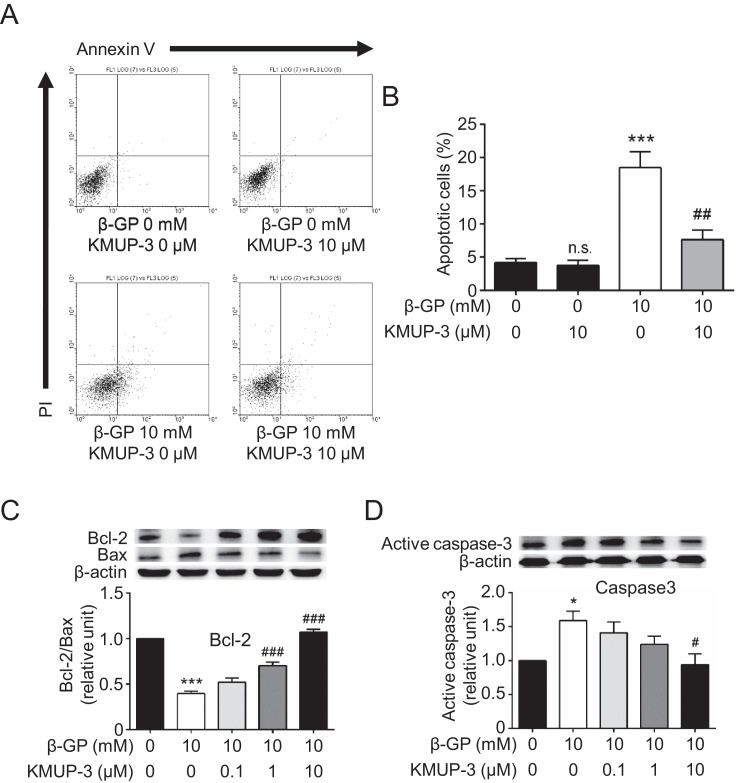
The effects of KMUP-3 on VSMC apoptosis during *In vitro* calcification. Primary rat VSMCs were incubated with KMUP-3 for 1 h before addition of β-GP for 48 h. (A) Representative flow cytometry. (B) Apoptotic cell percentage (*n* = 5). (C) Bcl-2/Bax level (*n* = 5). (D) Active caspase-3 level (*n* = 5). *p<0.05, ***p<0.001, n.s *p*>0.05 compared with the β-GP-negative, KMUP-3-negative group. #*p*<0.05, ##*p*<0.01, ###*p*<0.001 compared with the β-GP-only group.

We have demonstrated that KMUP-3 inhibits β-GP-induced VSMC calcification [1]. Herein, we evaluated whether KMUP-3 might inhibit VSMC apoptosis during VSMC calcification using VSMCs collected at 2 days. Flow cytometry revealed that the proportion of apoptotic cells in β-GP-incubated VSMCs was 18.5 ± 2.4% (Fig. 2A & B). However, in β-GP-incubated VSMCs pretreated with 10 μM KMUP-3, apoptotic cells were greatly reduced (7.6 ± 1.5%, *p*<0.01). KMUP-3 attenuated VSMC apoptosis during VSMC calcification. We investigated whether the Bcl-2/Bax ratio can be affected by KMUP-3 as the balance between anti-apoptotic Bcl-2 and pro-apoptotic Bax regulates VSMC apoptosis [1,2]. The Bcl-2/Bax ratio was lower in β-GP-incubated VSMCs than in control VSMCs, and the ratio was increased under pretreatment with 1 μM and 10 μM KMUP-3 (Fig. 2C). Also, the expression of activated caspase-3, which is activated in apoptotic cells [1,2], was increased in β-GP-incubated VSMCs (Fig. 2D), and the expression was significantly reduced by 10 μM KMUP-3.

**Fig. 3:**
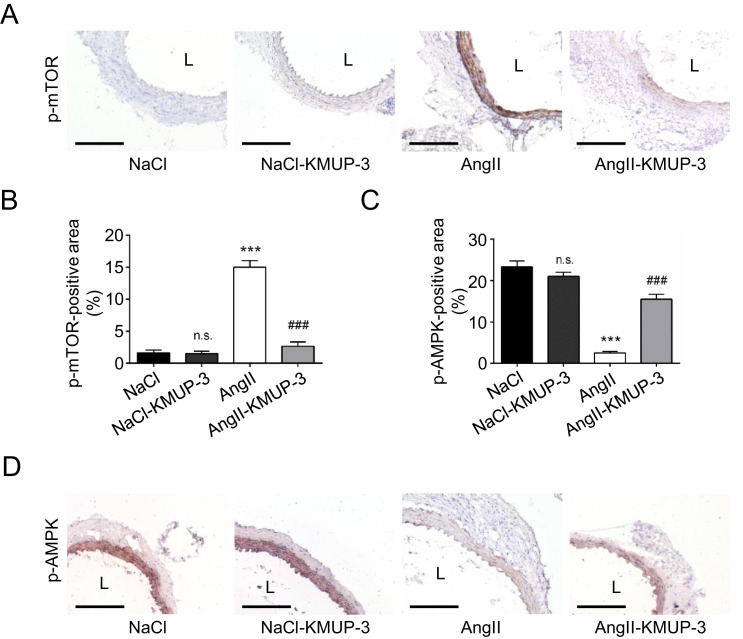
The effects of KMUP-3 treatment on p-mTOR expression and p-AMPK expression in the medial layer during AAA formation. (A) Representative microscopic images of p-mTOR staining. (B) p-mTOR-positive area (*n* = 6). (C) p-AMPK-positive area (*n* = 6). (D) Representative microscopic images of p-AMPK staining. L indicates lumen. All scale bars represent 100 μm. ***p<0.001, n.s *p*>0.05 compared with the NaCl group. ###*p*<0.001 compared with the AngII group.

**Fig. 4:**
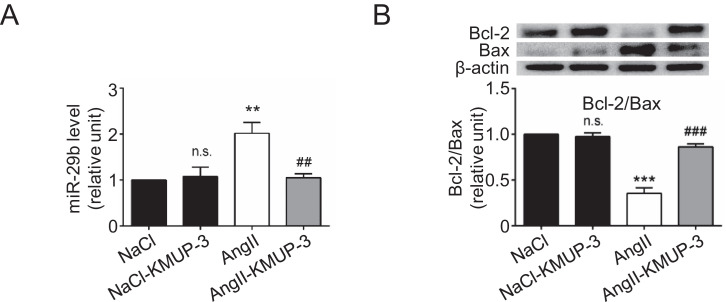
The effects of KMUP-3 treatment on the expression of miR-29b and ratio of Bcl-2/Bax during AAA formation. (A) miR-29b expression (*n* = 6). (B) Bcl-2/Bax ratio (*n* = 6). ***p<0.001, n.s *p*>0.05 compared with the NaCl group. ###*p*<0.001 compared with the AngII group.

We have demonstrated that KMUP-3 inhibits angiotensin II (AngII)-infused AAA [1]. Therefore, we investigated phosphorylated mTOR (p-mTOR) and phosphorylated AMP-activated protein kinase (p-AMPK) expression in the AAA specimens. On day 14, treatment with KMUP-3 led to reduced p-mTOR expression (Fig. 3A and B) and increased p-AMPK expression (Fig. 3C & D) in the medial layer, compatible with what we have observed *In vitro* [1]. As shown by quantitative real-time polymerase chain reaction (qRT-PCR), the aortic expression of miR-29b, a microRNA regulating apoptosis during aneurysm formation [3], was lower in the AngII-KMUP-3 group than in the AngII group (Fig. 4A). Also, KMUP-3 treatment increased the Bcl-2/Bax ratio (Fig. 4B), compatible with the *In vitro* findings (Fig. 2C).

**Fig. 5:**
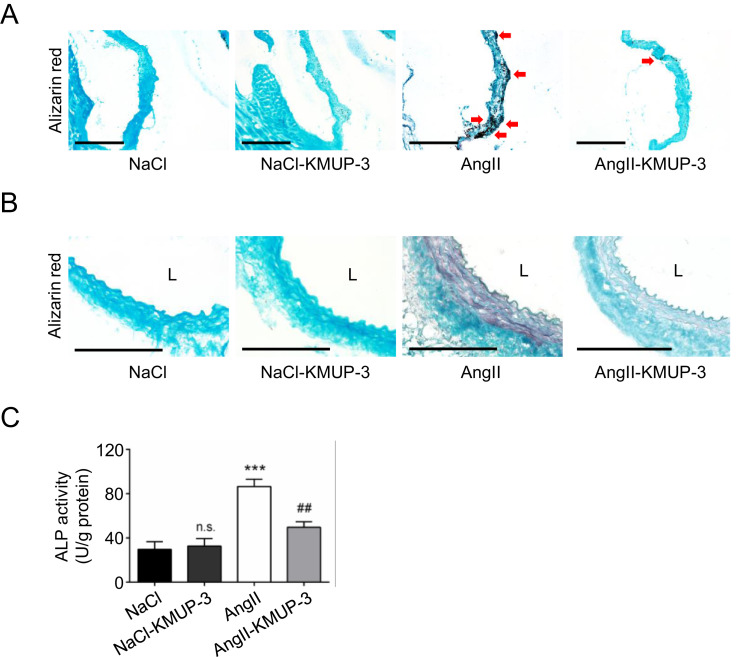
The effects of KMUP-3 treatment on aortic valve and vascular calcification. (A) Representative microscopic images of alizarin red staining in aortic valve samples. (B) Representative microscopic images of alizarin red staining in aortic samples. (C) ALP activity in the aortic wall (*n* = 6). Arrows indicate calcium deposits. L indicates lumen. All scale bars represent 100 μm. ***p<0.001, n.s *p*>0.05 compared with the NaCl group. ##*p*<0.01 compared with the AngII group.

Finally, whether vascular calcification can be altered KMUP-3 treatment was investigated. Compared with the AngII group, aortic valve calcification (Fig. 5A) and medial calcification in the aortic wall (Fig. 5B) in the AngII-KMUP-3 group were attenuated. Consistently, the alkaline phosphatase (ALP) activity in the aortic wall was increased in the AngII group, and the activity was lowered in the AngII-KMUP-3 group (Fig. 5C).

## 2 Experimental design, materials, and methods

### 2.1 Primary rat VSMC culture and *In vitro* VSMC calcification

The experimental details of *In vitro* VSMC calcification are described in our recent study [1].

### 2.2 Alizarin red S staining and calcium content in VSMC culture

Calcium deposition was analyzed using alizarin red S staining (Sigma-Aldrich, St. Louis, MO). After fixation with 75% ethanol, cells were stained with 2% alizarin red S solution for 1 h at 37 °C. The cells were incubated in 10% cetylpyridinium chloride solution (Sigma-Aldrich) for 30 min. The absorbance of the released alizarin red was measured at 540 nm. The calcium content in the cell lysates was evaluated using a QuantiChrom Calcium Assay kit (BioAssay Systems, Hayward, CA). The absorbance was measured at 612 nm using an automated microplate reader (SpectraMax 340PC384; Molecular Devices, Sunnyvale, CA).

### 2.3 ALP activity

To evaluate the activity of ALP in the cell culture, VSMCs were washed and lysed in Mammalian Protein Extraction Reagent (M-PER; Pierce, Rockford, IL). Samples were incubated in 0.1 M NaHCO3/Na2CO3 buffer containing 0.1% Triton X-100, 2 mM MgSO4 and 6 mM 4-nitrophenyl phosphate for 1 h at room temperature. The reaction was terminated by addition of 1 M NaOH. To evaluate the activity of ALP in aortic homogenates, the alkaline phosphatase activity colorimetric assay kit (BioVision, Milpitas, CA) was used based on the manufacturer's instructions. The ALP activity was determined by the absorbance at 405 nm.

### 2.4 Cell viability assay

The MTT assay (Sigma-Aldrich) was performed to assess VSMC viability. In brief, MTT (0.5 mg/ml) was added into the medium for 1 h. The culture medium was removed. Subsequently, the cells were dissolved in isopropanol and shaken for 15 min. The amount of MTT formazan product was quantified using an ELISA reader (Dynex Technologies, Berlin, Germany) at absorbances of 540 nm and 630 nm.

### 2.5 AngII-infused AAA model and KMUP-3 treatment

Treatment of KMUP-3 in the AngII-infused AAA model and other experimental details, including histological analysis, qRT-PCR and western blot analysis, are described in our recent study [1]. The whole study was approved by Institutional Animal Care and Use Committee of Kaohsiung Medical University (Number: 104,231) and conformed to the Guide for the Care and Use of Laboratory Animals published by the National Institutes of Health (NIH Publication #85–23, revised 1996).

### 2.6 Statistics analysis

Statistical analyses were performed using Prism 6 (GraphPad Software, San Diego, CA). All data were expressed as mean ± SEM. One-way analysis of variance followed by post hoc analysis (Bonferroni test) was used and a *p*<0.05 was considered statistically significant.

## Appendix Supplementary materials

Image, application 1
